# Building an International One Health Strain Level Database to Characterise the Epidemiology of AMR Threats: ESBL—AmpC Producing *E. coli* as An Example—Challenges and Perspectives

**DOI:** 10.3390/antibiotics12030552

**Published:** 2023-03-10

**Authors:** Sara Perestrelo, Ana Amaro, Michael S. M. Brouwer, Lurdes Clemente, Ana Sofia Ribeiro Duarte, Annemarie Kaesbohrer, Renata Karpíšková, Vicente Lopez-Chavarrias, Dearbháile Morris, Deirdre Prendergast, Angela Pista, Leonor Silveira, Magdalena Skarżyńska, Rosemarie Slowey, Kees T. Veldman, Magdalena Zając, Catherine Burgess, Julio Alvarez

**Affiliations:** 1 Department of Biological Safety, German Federal Institute for Risk Assessment, 10589 Berlin, Germany; 2 Laboratory of Bacteriology and Micology, National Institute of Agrarian and Veterinary Research, National Reference for Animal Health, 2780-157 Oeiras, Portugal; 3 Department of Bacteriology, Host Pathogen Interaction & Diagnostics, Wageningen Bioveterinary Research, Part of Wageningen University & Research, 8221 Lelystad, The Netherlands; 4 National Food Institute, Technical University of Denmark, 2800 Kongens Lyngby, Denmark; 5 Veterinary Public Health and Epidemiology, University of Veterinary Medicine, 1210 Vienna, Austria; 6 Department of Public Health, Medical Faculty, Masaryk University, 625 000 Brno, Czech Republic; 7 VISAVET Health Surveillance Centre, Universidad Complutense, 28040 Madrid, Spain; 8 Antimicrobial Resistance and Microbial Ecology Group, School of Medicine, University of Galway, H91 TK33 Galway, Ireland; 9 Backweston Laboratory Campus, Department of Agriculture, Food and the Marine, W23 X3PH Celbridge, Ireland; 10 National Reference Laboratory for Gastrointestinal Infections, Department of Infectious Diseases, National Institute of Health Doutor Ricardo Jorge, Avenida Padre Cruz, 1649-016 Lisbon, Portugal; 11 Department of Microbiology, National Veterinary Research Institute, 24-100 Pulawy, Poland; 12 Food Safety Department, Teagasc Food Research Centre Ashtown, D15 DY05 Dublin, Ireland; 13 Department of Animal Health, Faculty of Veterinary Medicine, Universidad Complutense, Avda. Puerta de Hierro S/N, 28040 Madrid, Spain

**Keywords:** *Escherichia coli*, extended-spectrum beta-lactamase (ESBL), AmpC beta-lactamase (AmpC), One Health, monitoring

## Abstract

Antimicrobial resistance (AMR) is one of the top public health threats nowadays. Among the most important AMR pathogens, *Escherichia coli* resistant to extended spectrum cephalosporins (ESC-EC) is a perfect example of the One Health problem due to its global distribution in animal, human, and environmental sources and its resistant phenotype, derived from the carriage of plasmid-borne extended-spectrum and AmpC β-lactamases, which limits the choice of effective antimicrobial therapies. The epidemiology of ESC-EC infection is complex as a result of the multiple possible sources involved in its transmission, and its study would require databases ideally comprising information from animal (livestock, companion, wildlife), human, and environmental sources. Here, we present the steps taken to assemble a database with phenotypic and genetic information on 10,763 ESC-EC isolates retrieved from multiple sources provided by 13 partners located in eight European countries, in the frame of the DiSCoVeR Joint Research project funded by the One Health European Joint Programme (OH-EJP), along with its strengths and limitations. This database represents a first step to help in the assessment of different geographical and temporal trends and transmission dynamics in animals and humans. The work performed highlights aspects that should be considered in future international efforts, such as the one presented here.

## 1. Antimicrobial Resistance as a Public Health Concern

Antimicrobial resistance (AMR) was identified as one of the top ten global public health threats according to the World Health Organization (WHO) [1], and represents a major challenge with extensive health and socioeconomic implications. In 2019, drug-resistant diseases were estimated to be responsible for at least 700,000 deaths globally per year, a figure that could potentially increase to 10 million deaths globally every year by 2050 [2]. Furthermore, more recent estimates suggest that 1.27 million deaths could be solely attributed to antibiotic-resistant bacterial infections [3]. Infections due to multidrug-resistant bacteria (MDR) are becoming more relevant, and are associated with an increased risk of complications, higher hospitalisation rates, increased healthcare costs, loss of productivity, and increased mortality. Estimates of additional annual healthcare costs, derived from these infections in the EU, are at least EUR 1.5 billion [4].

AMR is defined as the ability of microorganisms to become resistant to an antimicrobial to which they were initially susceptible and is a consequence of the natural selection of genetic mutations or acquired resistance genes via horizontal gene transfer. Although it is a natural phenomenon of microbial communities, AMR has emerged as a global health crisis due to the imprudent use and overuse of antimicrobials in the medical, veterinary, and agriculture sectors [5,6,7,8].

The misuse of antimicrobials, poor sanitation conditions, and inappropriate practices in healthcare settings or the food production chain create an enormous selective pressure on pathogenic and commensal bacteria, favouring the transmission of resistant bacteria [7]. Moreover, the environmental resistance gene pool plays an important role in this complex multi-factorial event on the epidemic increase in AMR [7,9].

Nowadays, difficult-to-treat human infections are commonly associated with MDR bacteria resistant to antimicrobials that are often considered first-line drugs for empirical therapy of severe infections, such as fluoroquinolones and β-lactam antibiotics. The impact of AMR in therapeutic success can be particularly severe when affecting vulnerable patients, such as children or immunocompromised individuals [10]. Of note, six leading human pathogens (*E. coli*, Methicillin-resistant *Staphylococcus aureus* (MRSA), *K. pneumoniae, Streptococcus pneumoniae*, *Acinetobacter baumannii,* and *Pseudomonas aeruginosa*), account for more than 70% of all deaths attributable to AMR [3]. These bacterial species, along with the genetic mechanisms mediating their phenotypic resistance, can also be found occasionally in livestock [11]. Therefore, the increase in resistance to antimicrobials in zoonotic pathogens and commensal bacteria, including last-resort drugs, such as colistin, is an important challenge for human medicine since it can lead to untreatable severe infections caused by Enterobacterales, among other bacterial families [9]. *Escherichia coli* is one of the six leading pathogens associated with resistance, with third-generation cephalosporin-resistant *E. coli* being among the most important pathogen-drug combinations responsible for deaths [3].

## 2. The One Health Paradigm for AMR: Extended-Spectrum β-Lactamase (ESBL) and AmpC β-Lactamase-Producing *E. coli*

Only a holistic view on the nature of AMR mechanisms and their sources and transmission routes in different ecological niches can help in addressing its spread and effectively combatting any further increase. As a global multifaceted and multi-layered phenomenon, AMR underlies the One Health paradigm [6,12,13], implying that human and animal health and the environment are interdependent [14].

Indeed, the problem of increasing AMR is not limited to bacteria in human and animal populations. Resistance mechanisms have also been reported among microorganisms present in the environment [7,14]. Therefore, environmental contamination with drug residues from hospital wastewater, municipal sewage, livestock farming, and agricultural waste can be selected for resistant bacteria. Other pharmaceuticals, such as biocides, antiparasitic, pesticides, and even metal ions may also affect the environmental microbiome. Moreover, these substances have been shown to influence horizontal AMR gene transfer, even between different bacterial genera [6,7].

There is still a limited understanding of the frequency of transmission of resistance between livestock and humans and, more importantly, of its impact on the incidence of infections with AMR bacteria in humans [15,16]. Even though multiple studies looking into the distribution of AMR bacteria and AMR genes in animals and food have been conducted, the pathways of transmission of AMR along the food chain are not well understood [17].

*E. coli*, a bacterial species present in the intestinal microbiota of mammals and birds, has often been used as an indicator of AMR dynamics in animals, humans, and the environment due to its ubiquity, frequent exposure to antimicrobial pressure, and genomic plasticity. According to Loayza et al. [18], the transmission of resistant commensal *E. coli* or AMR genes from *E. coli* from domestic animals to humans may occur frequently, but is difficult to prove. In addition, the diversity of *E. coli* clones and the turnover rate of these clones in the digestive tract hampers the identification of relationships between strains from domestic animals, animal products, and humans [18].

Among the most important emerging AMR threats is the global dissemination, and increasing prevalence, of plasmid-borne extended-spectrum β-lactamases (ESBLs) in *Enterobacterales*, a public health concern of particular relevance, since resistance to β-lactams limits the choice of effective antimicrobial therapies [19]. Since their discovery in the early 1980s, they have disseminated worldwide and are now widespread in *Enterobacterales* isolated from hospital-associated and community-acquired infections [20].

ESBL enzymes belong to the Ambler class A of β-lactamases, hydrolyse most penicillins and cephalosporins, namely, third- and fourth-generation cephalosporins, and are inhibited by β-lactamase inhibitors [21]. The most predominant enzyme families within this Ambler class A are represented by TEM-ESBLs, SHV-ESBLs, and CTX-M-ESBL enzymes. CMY is a common member of the plasmid-mediated AmpC Ambler class C, which does not hydrolyse fourth generation cephalosporins, is active on cefoxitin, and is not inhibited by clavulanic acid. The Bush-Jacoby nomenclature classifies β-lactamases into 17 functional groups based on their molecular and biochemical properties [21]. In this latter classification, the ESBLs TEM, SHV, and CTX-M are in Group 2 serine β lactamases, while CMY (AmpC) is a member of the Group 1 cephalosporinases.

In general, the presence of ESBLs/AmpC is a complicating factor for the treatment of patients with serious infections, since ESBL-producing bacteria are frequently MDR, often including resistance to fluoroquinolones [22]. It is clear that ESBL and AmpC producing *E. coli* (from here on called extended spectrum cephalosporin resistant *E. coli* or ESC-EC) infections place a burden on health-care systems, but there are inconsistencies in the method for defining the burden of illness of ESC-EC and other *Enterobacterales* in hospital settings [23]. Intestinal colonisation by ESC-EC and its association with community acquired MDR infections is of great concern; an increasing prevalence of ESBLs has been observed in the human gut microbiota in both healthy and diseased members of the community, and a recent study estimated a global eight-fold increase in the intestinal carriage rate of ESC-EC in the community over the past two decades [24]. Co-carriage of ESBLs within households were frequently observed in a systematic literature search, suggesting that interfamilial acquisition contributes to the spread of ESBLs in the community [25].

In parallel with the increasing incidence in humans, ESC-EC are more frequently reported in livestock, the food chain, and companion animals. As a consequence of this ongoing dissemination of ESBLs in domestic animals, wildlife [26], and the environment [27], they are considered as reservoirs and vehicles for the spread of ESBLs. In a recent study, the molecular characteristics of ESBL isolates from humans, animals, and the environment indicated a multi-directional spread of ESBL genes [28]. A follow-up study of the same dataset demonstrated that humans are the main source of community-acquired ESC-EC carriage, but this is unlikely to be self-maintaining without transmission to and from non-human sources [29]. These studies demonstrate the complexity of the dynamics involved in the transmission of ESC-EC and emphasise the need for ongoing efforts to identify scientific-based intervention measures to tackle their spread.

In general, an effective response to the increase in AMR (in particular, ESC-EC) requires international cooperation and common solutions to tackle the problem [13]. Therefore, only comprehensive and far-reaching research may help in identifying differences and similarities in AMR dynamics in different parts of the globe along with the mechanisms behind them, which in turn, can allow for the design of measures targeting these mechanisms. Above all, this requires global data sharing, which could be achieved by building platforms to exchange relevant information (e.g., AMR profiles, genetic determinants for resistance, virulence, typing, plasmid profiles, available sequences) on AMR strains from all One Health domains [14].

The benefits of analysing a large amount of data from different regions and preferably research areas include improved recognition of possible threats, sources, and vectors associated with the dissemination of resistant bacteria. It would also facilitate the detection of emerging resistant microbes and new mechanisms with the potential for worldwide expansion. Furthermore, considering geographical differences is of great importance in identifying potential factors that may affect the global spread of resistant microbes. It is worth emphasising that the effectiveness of efforts taken by individual countries to reduce AMR varies greatly, mostly due to the different dynamics in the management of activities to control AMR.

## 3. Factors Influencing the Epidemiology of ESC-EC That Should Be Considered in Surveillance

Several factors can influence the epidemiology of ESC-EC, or the results obtained from data describing its occurrence in different sources, and these should be accounted for when gathering data across countries in a common database. Among these factors are (1) the characteristics of the countries involved regarding, for example, climate, geology, or sanitation facilities, (2) the sampling strategies and the antimicrobial susceptibility testing methods used, (3) the timeframe of data collection, which should be considered in the context of the application of interventions, e.g., for antimicrobial usage reduction within that period, (4) the number, relative importance, and characteristics of ESC-EC reservoirs sampled within each country, including, for example, the characterisation of the livestock production systems, and (5) the number of *E. coli* isolates included per ESC-EC reservoir per time period within each country.

The distribution of ESC-EC and the frequency of appearance of specific ESBL types vary widely across different geographical areas globally and within the EU, suggesting that certain isolates expressing specific enzyme types are better adapted to some environments and geographical areas [30]. CTX-M, SHV, and TEM are the most common ESBL types identified in clinical cases in Europe and elsewhere, with CTX-M outnumbering the other two types in cases reported every year [31]. A higher proportion of ESC-EC positive samples in food-producing animals compared to food products has been described [32]. Regarding the geographical origin of the samples, within the EU, the highest levels of ESC-EC are reported in people in Southern and Eastern countries on a yearly basis [33]. This occurrence could be related to the higher consumption of these drugs, along with co-selection pressure due to the use of other antimicrobials and biocide substances, as reflected by higher sales numbers, associated with less strict antimicrobial usage policies in these countries [34].

*E. coli* isolates selected for ESBL monitoring are generally sampled differently from humans, animals, food, and the environment. While those from animals, food, and the environment usually represent commensal, non-pathogenic strains, human isolates most often originate from clinical samples of urinary or bloodstream infections. This difference must be accounted for when utilising data for ESC-EC source-attribution, or to assess the risk of transmission of ESC-EC between animals and humans. Whenever available, data from asymptomatic human carriers should be included [29].

Although national monitoring programmes generally try to account for representativeness in sampling and ensure harmonised testing, it may be difficult to follow the development and impact of various targeted interventions implemented in individual countries at different points in time. For example, interventions targeting the use of antimicrobials at the farm level have resulted in a decrease in resistance [35], and several countries have implemented interventions using different approaches and times [36]. When selecting data from an international dataset for a comparative study, the period considered may thus encompass the implementation of various interventions, changes in usage patterns, and the introduction of regulations at a national level. Therefore, it is important to be aware of any actions that may have had an impact on the data at hand and whether they should be considered as confounders or as explanatory variables.

In conjunction with the sampling of animals and meat from the major livestock sectors, other sources should be considered as potential reservoirs for transmission to humans, including (1) the environment through the collection of samples of sewage (a hotspot for horizontal AMR gene transfer) [37] and wild animals, (2) ground and surface water used for drinking and recreational purposes, (3) samples from smaller livestock production sectors, such as small ruminants, laying hens, ducks, rabbits, and farmed fish and shellfish, (4) imported food that may not be included in the sampling strategy on food products, and (5) samples from fresh fruit and vegetables. The additional sampling of healthy humans and companion animals [38] would also provide a cross-section of the general population. For these other sources, the current sampling numbers, sampling strategies, and methods are diverse. Available datasets are often collected for academic and research purposes and, as a result, these activities are frequently point prevalence studies, rather than the continuous monitoring that is performed in the major livestock sectors. This lack of harmonisation makes it more difficult to compare the distribution of ESC-EC between sectors, both nationally and internationally. Nonetheless, the inclusion of isolates collected through these studies does allow for qualitative comparisons between these isolates in terms of, e.g., presence of specific gene types, both nationally between putative reservoirs and between countries. While these comparisons can include the detection of co-resistance from phenotypic studies, building the capacity to determine the genetic distance between isolates in genomic studies and detecting the cross-over between reservoirs and across countries is imperative to identify the most likely sources for the human population and help in designing strategies for coordinated interventions in the future.

Considering the complex epidemiology of ESC-EC transmission in the One Health context, a database for establishing the most likely sources of infection for the general public in the EU should ideally include humans, food producing animals and retail food samples, pets and environment samples (fresh drinking water, surface water, sand/soil, sewage, and wildlife) [34,39]. Regarding animal sources, the prevalence of ESC-EC in food producing animals and food products is variable within EU countries, with countries reporting low to very high levels, and values vary within the same animal production cycle [32]. Ideally, these differences should be considered in the design of the sampling strategies, in order to generate a representative database on ESC-EC, in line with the EU guidelines for sampling at slaughterhouses, retail outlets, and border control posts for ESC-EC monitoring purposes [40]. Therefore, sampling should consider country-specific prevalence estimates from the previous year and geographic species-specific throughput variations, as indicated in EU technical guidance reports [32,41,42]. The monitoring at border posts is particularly important on consignments from other regions of the world (e.g., Asia, South America), as the levels of ESBLs reported in these regions are higher than in Europe [30,43]. Farms/fish farms, zoos, sewage treatment plants, and care home workers, as well as food handlers and travel associations, along with healthy humans, should be taken into consideration whenever possible [30,31].

## 4. Existing ESC-EC Surveillance Programs: Strengths and Weaknesses

Since 2014, the EU legislation has described the sources in which member states should monitor and report ESBL and AmpC producing *E. coli*, including broilers, turkeys, fattening pigs, and bovine animals under 1 year of age [44]. From these species, samples are obtained from both caecal content at slaughterhouses (for member states with >10,000 tonnes of a given species slaughtered per year) and from fresh meat at the retail level (except for turkey), whilst samples of fresh meat at border control posts have been added since 2021 [40]. Sampling and reporting on these four livestock species occur in alternating years, where broilers and turkeys are combined, as well as fattening pigs and bovine animals. As monitoring occurs on equal numbers per country (with some exceptions for countries that produce low amounts of specific species) and sampling strategies are harmonised amongst countries, bias is limited for these sources, although only a limited correction is included for the size of the production sectors in each country, and thus the sampling fraction may differ between member states. The harmonised nature of the sampling activities, along with the use of centralised protocols for the isolation of ESC-EC from the samples provided by the European Reference Laboratory for antimicrobial resistance (available at https://www.eurl-ar.eu/protocols.aspx, accessed on 15 January 2023), enable the extensive cross-country comparisons that are carried out [42].

Routine specific monitoring of susceptibility patterns in ESC-EC programs in the EU are based on the use of selective media supplemented with cephalosporins and on broth microdilution using two sequential antimicrobial panels according to the guidelines provided by EFSA, in order that the second panel allows for specific phenotypic characterisation of presumptive colonies of ESBL (and AmpC or carbapenemase)-producers detected with the first panel [45]. Results from these antimicrobial panels are interpreted according to the breakpoints recommended by EUCAST [46] and implemented in EU legislation (Commission Implementing Decision (EU) 2020/1729). In this framework, all member states currently have phenotypic results based on harmonised and well documented methods. As an alternative to phenotypic testing, it is now possible at the EU level to choose to report certain acquired resistance genes and chromosomal point mutations responsible for ESBL phenotypes based on whole genome sequencing (WGS) [40]. The integration of next generation sequencing methods in routine monitoring, and sharing whole genome sequences across countries, institutions, and sectors carries a new set of concerns despite the harmonised protocols and proficiency testing already available [47]. To share genomic data in an international, cross-sectoral collaboration, it is important to report and, whenever possible, harmonise the methods used for DNA extraction, library preparation, DNA sequencing, and bioinformatics analysis [48]. Nevertheless, the very high correlation observed between phenotype (i.e., minimum inhibitory concentration data) and genotype (i.e., presence/absence of genetic mechanisms predicted to confer resistance) demonstrates the potential usefulness of molecular-based methods for AMR monitoring [49].

The genetic mechanisms and the transmission of ESC-EC have been frequently investigated by PCR targeting specific genes [50]. In contrast to PCR, however, WGS offers the possibility to investigate all known ESBL genes contained in a genome and retrospectively investigate sequences for the presence of novel genes [51]. Moreover, WGS is more scalable than PCR, less cumbersome, and has a higher throughput. Therefore, it would be preferred as the source of genomic information in the context of large-scale sharing of monitoring data, although the use of standardised methods (e.g., for screening genomes against a harmonised database of resistance genes) would be required, in order to compile data from multiple sources.

Significant knowledge gaps exist in our understanding of the complex transmission dynamics of ESC-EC in and between animals, humans, and the environment, and studies incorporating traditional antimicrobial susceptibility testing alongside WGS could be useful in filling those gaps [29,52]. These studies on transmission pathways are dependent on the availability of a comprehensive database of high-quality sequences and corresponding phenotypic data [53].

WGS is a useful and accurate tool for predicting the AMR phenotype [54]; however, its value depends on the quality of the sequences used for downstream analysis [55,56]. The functional annotations provided by users when submitting to databases, such as the European Nucleotide Archive (ENA), can be affected by inaccuracies and the lack of harmonisation of pipelines used to detect resistance genes [55].

To date, most research projects and surveillance programmes have concentrated on AMR in bacteria from human and food animal sources [52], while relatively few have included bacteria from the environment [52,57], companion animals [58], or diseased animals [59]. Consequently, these sources are under-represented in databases of whole-genome sequences, which limits the understanding of the epidemiology of ESC-EC infection and the scope of source attribution studies. In addition, there may be gaps in the metadata accompanying sequences or issues with the standardisation of metadata categories.

The International Nucleotide Sequence Database Collaboration (INDSC) [60] partners include the DNA Data Bank of Japan (DDBJ), the National Centre for Biotechnology Information Genbank (NCBI), and the European Nucleotide Archive (ENA). The INDSC is a collaborative effort between Japan, the USA, and Europe to collect and disseminate databases containing DNA and RNA sequences. These databases have some common data requirements during the submission process [61], and allow users to upload sequence data directly. The three databases exchange data daily; therefore, once the sequenced data become available in one of them, they are shared with the other two [62]. Nevertheless, there are differences between the databases in terms of data standard requirements. For example, the ENA database uses a community-developed reporting standard [63] system to validate the uploaded data. For the data to be validated by ENA, users must provide mandatory information regarding their metadata depending on the sample type they submit [64]. When submitting data to NCBI, users need to provide information regarding the project or initiative in which the samples were collected, metadata regarding each isolate (for example, the collection date), and raw sequence data to be accepted [65]. One limitation of the current open-source databases is that submitters are responsible for the content and accuracy of their data, as curators do not supervise this process.

These limitations are also applicable to linked webtools, such as Resistome Tracker [66], which allows the user to explore recent submissions of AMR *E. coli* to NCBI. A similar interactive tool is the Surveillance Atlas of Infectious Diseases [67] from the ECDC, which contains information from country reports sent to the European AMR Surveillance Network (EARS-Net). This interactive database allows the user to visualise the prevalence and distribution of ESC-EC in the human population, but only contains data from 30 countries from the EU/EEA, and only phenotypic data are accessible. A strength of this tool, however, is that the data are reliable since the ECDC curates the data before publishing it.

Current databases contain large numbers of sequences—over 200,000 *E. coli*/*Shigella*, in the case of the NCBI Pathogen Detection Isolate Browser, which originate from all over the world, allowing for international comparisons to be made. However, since WGS is a relatively new and expensive technology, certain regions may be underrepresented; most isolates in the *E. coli*/*Shigella* NCBI database come from the United States of America (41%), followed by the United Kingdom (14%) and Australia (3%). Furthermore, the sampling location was not supplied for a large portion of sequences (15%), restricting their potential use.

The composition of repositories may also be limited by a reluctance of some institutes to share sequences and accompanying metadata due to concerns regarding their obligations under data protection legislation and potential reputational risk to food producers that might be implicated in disease outbreaks or foodborne AMR spread. Larger databases, such as INSDC are open access [68], while others, such as the ENA, have both open and private repositories with access limited to authorised users [55]. The planned database of ESBL genotypes from the harmonised AMR monitoring programme in animals and food throughout the EU [40] will have closed access, or contain information on genes as opposed to genome sequences due to sensitivities regarding data sharing [42].

The inclusion of phenotypic data alongside details on the resistome of individual isolates is important since the phenotype may be caused by multiple mechanisms that are yet undescribed, or the minimum inhibitory concentration of the isolate may be below the clinical breakpoint, and therefore, not associated with treatment failure in vivo. Repositories, including the Pathosystems Resource Integration Centre (PATRIC) database, the Sequence Repository Archive (SRA) of NCBI, and the AMR data hub developed around the ENA, have addressed this issue and allow for the submission of antimicrobial susceptibility (AST) data alongside genome sequences [53]; however, the number of genomes contained in these databases is small to date.

## 5. The DiSCoVeR ESC-EC Database

To assess the potential of generating a useful database with data from isolates from multiple sources (including human, animal, and environmental samples), the One Health European Joint Programme funded the DiSCoVeR (“*Discovering the sources of Salmonella, Campylobacter, VTEC and antimicrobial resistance”*) project (https://onehealthejp.eu/jrp-discover/, accessed on 15 January 2023). This project, including 19 partners from 13 countries, aims at improving the current understanding on the dynamics of transmission of some of the major zoonotic threats in the EU by considering both traditional (livestock, food) and non-traditional (wildlife, companion animals, environment) sources in a multidisciplinary framework based on existing and newly developed source attribution models. In this context, ESC-EC was selected as a working model to assess the potential of DiSCoVeR to generate new evidence that could help in better understanding the main sources and mechanisms of transmission of AMR threats between the One Health compartments. Partners were requested to share the available strain-level information on ESC-EC obtained from human, animal, food, and environmental sources originating from their research and surveillance activities, with 13 partners belonging to eight countries contributing to this task.

### 5.1. Countries, Data Sources, and Isolate Characteristics

The DiSCoVeR ESC-EC database gathered a total of 10,763 isolates, which were submitted by public health, animal health, and research institutes located in the Czech Republic, Denmark, Germany, Ireland, the Netherlands, Poland, Portugal, and Spain. The provided information included the isolate ID, year of isolation, institute (and country where the institute was located), country of origin of the sample from which the strain originated, source information (including source group, subgroup, and sample type), whole genome sequence status, AMR typing (including method and minimal inhibitory concentration), and ESBL genes presence (Table 1 and Supplementary Data S1 for further information). The isolates were collected between 2013 and 2020, with 2016 being the year with most isolates (*n* = 2278, 21%), followed by 2018 (*n* = 1708, 16%) and 2017 (*n* = 1593, 15%). The isolates were organised into seven source subgroups: Livestock (*n* = 9444, 88%), human (*n* = 711, 7%), environment (*n* = 470, 4%), wild animals (*n* = 103, 1%), pets (*n* = 26, <1%), fruit/vegetables (*n* = 7, <1%), and zoo animals (*n* = 2, <1%).

In terms of individual countries, partners in Germany (*n* = 3955, 37%) provided the highest number of isolates, followed by the Netherlands (*n* = 2727, 25%), Spain (*n* = 2228, 21%), Denmark (*n* = 607, 6%), Ireland (*n* = 539, 5%), Portugal (*n* = 437, 4%), the Czech Republic (*n* = 151, 1%), and Poland (*n* = 119, 1%).

All countries, except for the Czech Republic, submitted data obtained through national monitoring programs performed according to the national and European legislation (Commission Implementing Decision 2013/652/EU). Most countries (Denmark, Germany, Ireland, Poland, and Spain) also sent data from additional research projects, which included uncommon sources, such as zoo animals, fruits, and vegetables. The Czech Republic only sent data collected from research projects.

The majority of isolates (*n* = 10,035, 93%) had been tested using antimicrobials included in the harmonised EU antibiotic panel for testing *Salmonella*/*E. coli* (EUVSEC, Sensititre™, Thermo Fischer, Waltham, MA, USA) [69]. Most isolates (9234, 86%) were tested by microdilution, with the remaining isolates being tested by disk diffusion (738, <1%) and agar dilution (63, <1%). For 96.9% of the isolates (8952 of the 9234 isolates tested using microdilution), minimum inhibitory concentration (MIC) data for the antimicrobials included in the second panel (EUVSEC2, Sensititre™, Thermo Fischer), allowing for the confirmation of the ESBL phenotype in presumptive ESBL producers (cefotaxime, ceftazidime, and clavulanate synergy test in combination with those two), were available. Out of these, the majority of the isolates had a confirmed ESBL phenotype (MIC > 1 mg/L for cefotaxime and/or ceftazidime and synergy between these antimicrobials and clavulanic acid, coupled with MICs below the ECOFFS for cefoxitin and meropenem, found in 7237 isolates, 80.8%) or ESBL + AmpC phenotype (same as before but with MIC > 8 mg/L for cefoxitin, observed in 528 or 5.9% of all isolates) according to EUCAST guidelines [46] and EFSA recommendations [70]. Most of the remaining isolates fell into the AmpC phenotype category (MIC > 1 mg/L for cefotaxime and/or ceftazidime and MIC > 8 mg/L for cefoxitin but negative results in both synergy tests with clavulanic acid, found in 1146 isolates, 12.8%), while 41 (0.5%) expressed other phenotypes.

Regarding the presence of beta-lactamase encoding genes, a total of 5416 (50.3%) isolates carried at least one *bla*_CTX-M_ gene, 1606 (14.9%) isolates had at least one *bla*_TEM_ gene and 898 (8.3%) isolates had at least one *bla*_SHV_ gene. Of note, several of these genes (e.g., *bla*_TEM-1_) do not confer an ESBL phenotype by themselves, and therefore, in some cases, additional ESBL-genes/chromosomal mutations would have had to be present (but were sometimes not reported), in order for the strains to express this resistance phenotype.

Among the 5416 isolates in which the presence of *bla*_CTX-M_ genes was reported, 56 (1.0%) contained more than one *bla*_CTX-M_ gene according to the information provided. The *bla*_CTX-M-1_ gene was the most common in the database (*n* = 2667, 49.2%), followed by *bla*_CTX-M-15_ (*n* = 1132, 20.9%) and *bla*_CTX-M-14_ (*n* = 375, 6.9%). Nevertheless, the degree of existing information regarding the presence of these genes was different depending on the isolate, as for 635 (11.7%) isolates, only information regarding their *bla*_CTX-M_ groups (e.g., *bla*_CTX-M-1_ or *bla*_CTX-M-9_ groups) was available.

For *bla*_TEM_-carrying isolates (*n* = 1606), *bla*_TEM-1_ (including variants, such as *bla*_TEM-1A_ or *bla*_TEM-1B_) was the most common (*n* = 1015, 63.2%), followed by *bla*_TEM-52_ (*n* = 544, 33.9%). There were also combinations with more than one *bla*_TEM_ gene (or gene variant) identified (e.g., *bla*_TEM-52_, *bla*_TEM-20_) in 85 isolates (5.3%), though some of these included variants of the same gene, and thus probably indicated the presence of a single specific gene. Of note, in one isolate, up to five *bla*_TEM_ genes were reported (*bla*_TEM-20_, *bla*_TEM-47_, *bla*_TEM-68_, *bla*_TEM-126_, and *bla*_TEM-207_). Six isolates did not have their *bla*_TEM-_ genes confirmed and were described as “*bla*_TEM-30_ or *bla*_TEM-99_” and “*bla*_TEM-163_ or *bla*_TEM-99_”.

Finally, the *bla*_SHV-12_ gene (*n* = 860, 95.7%) was the most common gene within the isolates that carried *bla*_SHV_ genes (*n* = 898), followed by *bla*_SHV-2a_ (*n* = 13, 2%) and *bla*_SHV-2_ (*n* = 9, 1%). A combination of more than one *bla*_SHV_ gene was found in one isolate (*bla*_SHV-12_, *bla*_SHV-129_).

The database contained information regarding AmpC genotypes in only 866 (8.0%) isolates, though some of these referred to chromosomal mutations in promoter/attenuator regions rather than to plasmid-mediated genes. For the latter, *bla*_CMY-2_ was by far the most commonly reported (480 out of 485 strains in which *bla*_CMY_ genes were reported).

Only a small number (*n* = 1348, 13%) of isolates in the database were analysed by WGS.

### 5.2. Limitations

The present database is a compilation of the existing data on ESBL genes in the European Union, and it may serve as a valuable resource for risk assessment studies, including source attribution models. It brings together data from a diverse range of sources and countries, enabling a more representative One Health focused analysis and providing a useful demonstration of the possibilities of this approach. Nevertheless, there are some limitations and challenges in terms of it usability. First, it lacks geographical representativeness: From the 27 member states of the European Union, only eight are represented, which limits possible comparisons between countries and regions. Second, there is a large gap between the number of available isolates in each sector. The livestock sector is well represented, with most isolates belonging to this category, but pets and wildlife animals are underrepresented. Moreover, the human and the environmental sectors have comparably lower numbers of isolates than the livestock sector. Human data are only available in four out of ten countries (Germany, *n* = 463; Netherlands, *n* = 230; Portugal, *n* = 17; Czech Republic, *n* = 1) and environmental data are only available from Ireland (*n* = 424) and the Czech Republic (*n* = 46). These data gaps between sectors pose challenges for source attribution modelling, given the fact that human data are crucial to develop these models, and that the One Health approach cannot be successfully achieved without using data from all three sectors (animals, environment, and humans).

Two countries (Denmark and the Czech Republic) included data (*n* = 204, 2%) from imported food and environmental samples from other countries. This can be misleading since the information regarding these isolates does not represent the country of reporting, but rather the country of origin of the samples. Data that referred to imported food from outside of Europe were not considered in our analysis. Nevertheless, isolates from imported food within Europe were included in the database. To avoid biased results, further studies and source attribution models should consider the country in which the sample was collected rather than the reporting country.

Additionally, the data provided by the different institutes were not described in a homogeneous way. The project partners had a shared template to complete the bacterial isolate information. Nevertheless, there were major differences in the level of detail provided in the database by the partners. This is especially observed in the way that ESBL genes were described (e.g., reporting CTX-M groups vs. specific gene types, such as *bla*_CTX-M-10_). Moreover, we found that some isolates had many combinations of the same ESBL gene, such as one isolate in which five different *bla*_TEM_ genes were detected (*bla*_TEM-20_, *bla*_TEM-47_, *bla*_TEM-68_, *bla*_TEM-126_, *bla*_TEM-207_), while in other isolates the specific identity of their ESBL genes was not confirmed (“*bla*_TEM-99_ or *bla*_TEM-163_”). The identification of multiple variations of the same gene may also arise from the software used to predict the presence of resistance genes if there are incomplete hits to the gene. This lack of harmonisation in the description of isolates, most likely related to the way the data were generated (i.e., use of PCRs for ESBL groups vs. use of WGS) or the purpose for which they were collected, is challenging when using the data for source attribution models, as it is hard to know which values were correctly validated/confirmed.

Another challenge was the existence of duplicate isolates, derived from mistakes while introducing the data, but also due to the existence of isolates sharing the same ID and originating from different institutes/countries. Moreover, a significant portion of isolates (*n* = 2797, 26%) did not have any information regarding their ESBL or AmpC genes, which indicates they cannot be used for source attribution modelling.

Furthermore, the database included isolates in which only non-ESBL genes (e.g., *bla*_TEM-135_) were detected. In addition, among the isolates in which data from both EUVSEC plates were available, some did not express the full ESBL-phenotype (i.e., MIC > 1 mg/L for cefotaxime and/or ceftazidime and synergy between these antimicrobials and clavulanic acid, coupled with susceptibility to cefoxitin and meropenem), and therefore, would fall into other phenotypical categories (in addition to AmpC-producing EC) based on the resistance to the β-lactams included in Panel 2 [70]. However, the inclusion of these non-ESC-EC would depend on the system, by which the isolates were retrieved and/or selected in each partner institution, which was heterogeneous (Supplementary Data S2).

## 6. Possible Applications for an International One Health Strain Level ESC-EC Database to Characterise Its Epidemiology

### 6.1. Source Attribution Models

Source attribution studies estimate the extent to which certain sources might be responsible for human cases related to zoonotic diseases [71]. These models use retrospective data from different sources, such as animals (e.g., livestock, pets, wildlife), the environment (e.g., wastewater, soil, rivers), and humans (e.g., nosocomial patients, international travel) to assess the similarity between the isolates in these sources and those originating from diseased humans.

There are different approaches to source attribution and one of the most popular is to use microbial subtyping data. Microbial subtyping techniques can include serotyping, phage typing, AMR profiles, or molecular techniques, such as WGS [72]. With this available information (serotypes, resistance types, or genotypes), source attribution models can be successfully generated. In the case of ESC-EC, specifically, data regarding ESBL and pAmpC genes, phylogenetic group and/or AMR profiles can be helpful for running these models [29,73].

In general, source attribution models require large and representative sample sets from each source, as these models only attribute illness to those sources from which isolates are available [71]. This can be a limitation of this method since the amount of available data for resistant bacteria varies greatly between countries and sectors. Nevertheless, when large amounts of data are available, having an international database can be helpful for comparing the main sources of infection in different countries or regions [74]. Moreover, it can help in identifying associations between the particular gene presence in given sectors or geographic regions.

The current ESC-EC database is a significant resource, which has the potential to be used for source attribution purposes once the data are cleaned and standardised. Given the fact that the number of isolates for human data is only representative for Germany and the Netherlands, a source attribution model using data from animals and humans from these two countries (as performed with a subset of the data from the Netherlands) [29] can be envisioned. Furthermore, the environmental data from Ireland can provide the opportunity to include this sector in a source attribution model. Since Germany, the Netherlands, Ireland, and Denmark are all located in Western Europe, a source attribution model, including data from these four countries could be useful in understanding the sources of ESC-EC in that region.

### 6.2. Assessment of Geographical and Temporal Trends

Information regarding resistant bacteria in humans, animals, and the environment over different regions and years can be used to assess geographic and temporal trends. The Surveillance Atlas of Infectious Diseases from the ECDC [67] is a good example of a tool that has benefited from large sets of data coming from various countries in Europe over the last 20 years. The presented maps and graphs are a reliable and comprehensive overview of trends in resistant bacteria in Europe. In this case, the database uses the total numbers of resistant (R), intermediate (I), and sensitive (S) isolates in a selection of antibiotics. Nevertheless, the Surveillance Atlas from ECDC does not include trends specifically for ESC-EC. The current DiSCoVeR ESC-EC database could be used to assess temporal and spatial trends specifically for ESBL genes and resistance profiles to antimicrobials, which are closely related to ESBL-producing bacteria in Europe for the period between 2013 and 2020.

### 6.3. Outbreak Investigation and Surveillance of Pathogens and Foodborne Disease

Another use of global databases for resistant bacteria can be for outbreak investigation and disease surveillance. These databases need to be continuously updated, in order that the potential cause of an outbreak can be quickly identified. Having the One Health approach in these databases is important, as bacteria responsible for human cases might have varied sources. Databases used for outbreak and surveillance purposes benefit from being combined with other digital tools that can analyse and compare the included data, for instance, by clustering bacteria with similar genotypes into phylogenetic trees. An example of an online portal that combines data collection and analysis is EpiPulse from the ECDC [75]. This platform enables the collection, analysis, and dissemination of surveillance data on infectious diseases and associated health issues, acting concomitantly as a database and a surveillance tool.

A potential benefit of future international databases that collaborate with analysis tools and include WGS data might be to facilitate the identification and tracking of foodborne illness due to imported food items and infections amongst international travellers. These databases and tools could also be used in hospitals and healthcare facilities through the collection, categorisation, and analysis of the most common bacterial genotypes amongst their patients. This way, hospitals could identify and track human cases with a nosocomial origin.

In terms of real-time surveillance, apart from an up-to-date database, it is also necessary to have supporting software that can aggregate, read, and interpret these data in real time.

## 7. Conclusions and Perspectives

The dynamics of AMR dissemination require the establishment of coordinated international and cross-sectoral collaboration, in order to implement common strategies against it. Therefore, research specialists from various fields and transdisciplinary studies must be involved to find an adequate solution. Although national actions, including surveillance of AMR organisms and proper antimicrobial stewardship, are essential to reduce the occurrence of resistant bacteria, being limited to local interventions may have only a negligible impact on the global AMR spread [76].

AMR gene abundance strongly correlates with socioeconomic, health, and environmental factors in various regions and continents. Therefore, improving education, sanitation, and health could potentially limit the global burden of AMR [77]. Moreover, preventive non-antimicrobial strategies, including timely vaccination, herd-specific biosecurity measures, proper nutrition and housing, may reduce the demand for preventive antimicrobial chemotherapy.

All these aspects highlight the importance of international and cross-sectoral research. To mitigate the increasing AMR, a broader global perspective that ensures the recognition and understanding of general trends in the dissemination of resistant bacteria in all One Health sectors is crucial and can only be supported through data sharing following the FAIR principles (findability, accessibility, interoperability, and reusability) [78] and coordinated analysis. The development of the DiSCoVeR ESC-EC database represents a first step in this regard, providing a database of over 10,000 bacterial isolates from eight countries, representing a myriad of sources. However, the development of the database has highlighted the challenges posed by this endeavour. The benefits of structured routine monitoring programmes are evident, as most isolates in the database are derived from these activities. These isolates are all collected and analysed in a comparable standardised way, although certain aspects (such as the determination of the specific genes conferring the ESBL phenotype) were not implemented in the same way in all countries. Furthermore, the data included represent a limited set of sources (i.e., four livestock species). The limited number of human isolates is likely a factor of the consortium composition and concerns data sharing, along with the limited monitoring of ESC-EC of human origin. Nevertheless, it also highlights the limitations associated with having different entities (agencies, ministries, or departments) in charge of the collection of data from the different compartments (animals/food/human) in most countries, data which are then typically stored in separate databases suited for these specific compartments (i.e., including information considered relevant for a given source) that are not routinely shared or synchronised. This, in turn, can hamper the ability to effectively combine data from the different sectors from the One Health perspective.

The addition of isolates originating from research projects enabled the consideration of a significantly broader range of sources, but their inclusion also brought a greater heterogeneity in the information included in the database, which poses analytical challenges. This heterogeneity may have arisen for a number of reasons, such as the use of local protocols or incomplete antimicrobial susceptibility testing due to a limited focus in the context of a specific project or budget constraints. To address the heterogeneity and support more complete cross-sectoral analysis, it would be beneficial to have a minimum set of guidelines/requirements for analysing and reporting ESC-EC to avoid possible biases, such as overrepresentation of certain regions/sources and repeated sampling of the same epidemiological units. This approach has been considered for the publication of quantitative PCR results [79]. The development of these guidelines would provide, particularly for research projects, a specific list of the requirements for the publication of results, which would enable their inclusion in broader analysis and comparisons with other studies on an international level, thereby benefiting the scientific community. Moreover, it would facilitate better integration of results from routine monitoring programmes with more point prevalence data originating from ad hoc research studies. The increased use of WGS for AMR analysis will facilitate these approaches, but again is challenged by the range of software and pipelines available, for example, the usage of different percentage identity criteria. Recent efforts have been made to address this through the provision of benchmarking datasets [80]. The development of the database described in the present work represents a concerted effort to bring together ESC-EC data from diverse sources to enable cross sectoral, One Health-focused analysis, and through its development identified challenges that need to be addressed. Nonetheless, the use of these harmonised approaches will greatly contribute to further understanding the dynamics of AMR transmission in and between different sectors and support the implementation of appropriate mitigation measures.

## Figures and Tables

**Table 1:** Sampling strategy and number of isolates per source and country submitting the information in the DiSCoVeR ESC-EC database.

Country	Environment	Human	Livestock	Pets	Wildlife	Others ^1^	Total	Sampling Years	Sampling Strategies ^2^
Czech									
Republic	46	1	104	0	0	0	151	2015–2020	OP
Denmark	0	0	607	0	0	0	607	2013–2020	NMP + OP
Germany	0	463	3429	0	59	5	3955	2013–2020	NMP + OP
Ireland	424	0	114	0	0	1	539	2015–2020	NMP + OP
Netherlands	0	230	2497	0	0	0	2727	2014–2020	NMP
Poland	0	0	98	2	19	0	119	2013–2020	NMP + OP
Portugal	0	17	368	24	25	3	437	2013–2020	NMP + OP
Spain	0	0	2228	0	0	0	2228	2014–2019	NMP + OP

^1^ Isolates from fruit/vegetables and from zoo animals. ^2^ Isolates collected through the National Monitoring Program (NMP) or within Other Projects (OP).

## Data Availability

The data from this study are presented in the text and Supplementary Materials.

## Supplementary Materials

The following supporting information can be downloaded at: https://www.mdpi.com/article/10.3390/antibiotics12030552/s1. Supplementary Data S1: DiSCoVeR ESBL-EC complete database; Supplementary Data S2: Source of data included from contributing partners. References [44,81,82,83,84,85] are cited in the Supplementary Materials.
