# Supplementary material for: Building an International One Health Strain Level Database to Characterise the Epidemiology of AMR Threats: ESBL—AmpC Producing E. coli as An Example—Challenges and Perspectives
Source: Antibiotics (Basel). 2023 Mar 10;12(3):552. doi: 10.3390/antibiotics12030552 (PMC10044432; doi:10.3390/antibiotics12030552)
Supplement: Supplementary file 1 [file antibiotics-12-00552-s001.zip › Supplementary data S2_revised.pdf]

Supplementary data S2: source of data included from contributing countries.

Czech Republic

The CZ ESC-EC database included isolates obtained from retail meat samples in the Czech Republic (n=35) during the sampling period 2017 -2019 and from the natural environment (n=18).

The majority of *E. coli* isolates originated from products from Europe (69 %; Czech Republic (9 isolates), Poland (6), Germany (5), France (2), not specified (2). Isolates from non-EU products (31%) originated from Asia (6) and South America (5). Out of 35 isolates, the majority were retrieved from poultry meat (40% turkeys and 17% broiler-chickens), followed by pork (14%), frog legs (11%), beef (9%), rabbit (6%), and mixed minced pork and beef (3%).

Isolates from the natural environment (n=18) were collected in the Czech Republic during the sampling period 2016 – 2020: 2016 (7 isolates), 2017 (5 isolates), 2019 (3 isolates), 2020 (3 isolates). Isolates originated especially from water ecosystems: surface freshwater (n=10), mud from the banks of rivers and ponds (n=6), moss with soil (n=1), and decaying vegetation near the brook (n=1).

The strain collection was obtained within a research project focused on monitoring of ESBL and *mcr*-mediated colistin resistance.

Denmark

The Danish database consists of 607 ESC-EC isolates obtained via selective culturing from faecal samples of broilers (122), cattle (39) and pigs (152) collected at slaughter and from beef meat (28), pork meat (32) and chicken meat (234) sampled at retail, according to the national resistance monitoring program in livestock and meat, following European legislation [44]. Isolates were retrieved between 2015 and 2020. Pork and beef meat were sampled in every odd year (2015, 2017, 2019), while pigs and cattle were sampled at slaughter in 2015 and 2019. Broilers and broiler meat were sampled in every even year (2016, 2018, 2020), and broiler meat was also sampled in 2019. The total number of isolates cultured in a year ranged from 17 (2017) to 189 (2018). Among all 294 isolates retrieved from meat, 45% originated from meat produced in Denmark (44% of pork samples, 46% of broiler meat samples, 43% of beef samples) and 55% originated from imported meat.

Germany

The German ESC-EC database contained 3,956 isolates. The majority of isolates were from the livestock sector (n=3,428, 87%), and from humans (n=464, 12%). The remaining isolates belong to wild animals (n=76, <1%) and fruit and vegetables (n=5, <1%). Out of the livestock isolates, 2113 (62%) were from poultry (broiler-chickens n=1115, 53%; turkey n=929, 44%; laying hens n= 58, 3% and other poultry n=11, <1%) followed by pig (n=734, 38%) and cattle (n=561, 26%). Also, the database included 11 (<1%) isolates from farmed fish and 7 (<1%) isolates, which originated from the livestock sector but without information from which animal species ("livestock_unknown").

The animal isolates were collected between the years 2016 and 2021 and analyzed at the German Federal Institute for Risk Assessment. The isolates were collected under the scope of CD652/2013 [1] and also within the national zoonoses monitoring program implemented in 2009 in Germany.

The 464 human isolates from Germany were sent to Ruhr University for surveillance purposes by different laboratories and clinics in Germany. Most isolates originated from clinical samples from patients hospitalized in German clinics. They were isolated from non-sterile matrices (n=390, 84%), such as wounds and rectal swabs, few originated from urine (n=11, 2%) and blood samples (n=4, <1%). The collection period was from 2013 to 2021, excluding the year 2019 in which no human samples were available.

Ireland

The collection of ESC-EC originated from several partner institutions contributing with isolates from different sources. Part of the ESC-EC which were cultured as part of official harmonised monitoring in Ireland originated from caecal content of healthy broilers (n=20) and pigs (n=19) and pork (n=15), chicken meat (n=15) and beef (n=6) sampled at retail. Isolates from samples such as milk, internal organs and swabs of clinically sick birds, pigs and calves were also included in the collection (n=17); these were recovered using non- selective isolation methods as part of veterinary diagnostic investigations.

Also included are 79 ESC-EC that were isolated from four different farm types that use natural wastewater management systems called integrated constructed wetlands (ICWs) for the treatment of animal waste. Selective media was used to screen for the presence of β-Lactamase producing organisms (ESBL/AmpC) as described by Prendergast et al. [81], and all were phenotypically confirmed using the Sensititre broth microdilution method.

Another subset of the collection of ESC-EC (n=345) were isolated from a range of environmental sources including seawater, lake water, river water and sewage as described by Hooban et al. [82], using Brilliance ESBL agar. Presumptive isolates were confirmed as *E. coli* using MALDI-TOF mass spectrometry. Antimicrobial susceptibility profiles for a range of antimicrobials were determined according to EUCAST guidelines, or CLSI where not available from EUCAST. ESBL production was confirmed using cefpodoxime alone and in combination with clavulanic acid.

Finally, 56 ESC-EC also included in the collection originating from research projects were isolated from pig faeces and the pig production environment on TBX agar containing 4 µg/ml cefotaxime [83]. Presumptive isolates were confirmed as *E. coli* using MALDI-TOF mass spectrometry. Antimicrobial susceptibility profiles for a range of antimicrobials were determined according to EUCAST guidelines, or CLSI where not available from EUCAST. ESBL production was confirmed using the Mast Group AmpC ESBL detection kit.

The Netherlands

The NL (WBVR) database contains information of 2,497 ESC-EC isolates obtained from faecal samples of livestock collected at slaughter in the period 2014 – 2020. This collection consists ESC-EC isolates retrieved from broilers (n=1053), ducks (n=14), laying hens (n=156), pigs (n=394) and cattle (n=880). During this period, ESC-EC isolates from broilers, pigs and cattle were collected each year, whereas isolates from laying hens were retrieved in the years 2014, 2016 and 2020 and isolates from ducks were only collected in 2016.

All ESC-EC isolates were obtained via selective culturing of faecal samples of livestock collected within the national resistance monitoring program in livestock and meat according to European legislation (Commission Implementing Decision 2013/652/EU) [1].

Poland

A total of 119 ESC-EC isolates from Poland were included in the database. The vast majority of these isolates were recovered from livestock samples (n=98, 82%) mostly from turkeys (n=33, 28%), broiler chickens (n=24, 20%) and pigs (n=26, 22%). Samples were collected at slaughter within 2013/652/EU: Commission Implementing Decision of 12 November 2013 during the years 2014-2020 [1]. Isolates from laying hens (n=4, 3%) and cattle (n=11, 9%) sampled as part of the multiannual national program (2017–2022) were also included.

The remaining ESC-EC were recovered from wild animals (wild boars, n=11, 9%; wild birds, n=6, 5%, hedgehog, n=1, 1%; and pets, n=2, 2%). Samples from wild animals were collected for several research purposes. Wild boar faeces (n=8) collected between 2013 and 2014 came from study on antimicrobial resistance in wild animals in Poland [84], and three from 2016 were collected within the European Union-funded EFFORT project (www.effort-against-amr.eu). ESC-EC from wild birds (n=6, 5%) were isolated during the years 2013-2018 for the purpose of the project on antimicrobial resistance in avian populations [85]. Two isolates from pets originated from reptiles that were sampled in 2013 for a research study.

Portugal

The Portuguese database comprised information from sequenced ESC-EC isolates recovered from animal (n=330, 70%), food (n=122, 26%) and human clinical (n=18, 4%) samples, collected between 2009-2021.

Among the animal isolates, 256 (78%) were retrieved from caecal samples collected at slaughter, under the scope of CD652/2013 [1], of which 114 (45%) were from fattening pigs, 87 (34%) from broilers, 44 (17%) from beef calves and 11 (4%) from fattening turkeys. The remaining isolates (n=74, 22%) were recovered from 34 (46%) pets (dogs, n=22; cats, n=7; birds, n=2; unknown, n=3), 32 (43%) wild animals (seagulls, n=21; wild birds, n=8, deer, n=1; wild boar, n=1; wolf, n=1), and eight (11%) zoo animals (dolphins, n=5; birds, n=2, carnivore, n=1). These samples were collected for bacteriological diagnosis and under the scope of research projects.

Regarding food isolates, the vast majority were recovered from meat samples (n=119, 98%) collected at retail between 2014-2020, under the scope of CD652/2013 [1]. Most samples were from broiler meat (n=62, 52%), followed by pork (25%, n=30) and beef (23%, n=27). The remaining isolates (n=3, 2%) were recovered from vegetables (n=2) and dairy products (n=1).

Human clinical isolates were received at the National Reference Laboratory, between 2013-2021, for the detection and characterization of pathogenic *Escherichia coli* and were recovered from faeces (n=11), urine (n=5), blood (n=1) and cerebrospinal fluid (n=1) samples.

Spain

The database comprised information from ESC-EC isolates retrieved mostly from faecal samples from livestock (broiler chickens, turkeys, pigs and cattle) (n=2029, 91%) collected at the slaughterhouse through the Spanish national AMR monitoring program, performed according to the national and European legislation [1]. The remaining 11% (n=199) consisted of isolates retrieved from meat samples from chickens, turkeys and pigs obtained from a regional monitoring program.

The sampling period spanned from 2014 to 2019, with roughly 500 isolates retrieved each year from 2014 to 2017, and about 200 and 75 isolates cultured in 2018 and 2019, respectively. Isolates from faecal samples were available for 2014, 2016 and 2018 in poultry, and in 2015,

2017 and 2019 for pigs and cattle. Isolates from broiler meat samples were retrieved every year during 2014-2019, while isolates from pork meat were cultured in 2016, 2017 and 2019, and from turkey meat only in 2019.

Out of 2228 isolates, the majority (64%) were from poultry (32% each broilers and turkeys), 23% from pigs and 13% from cattle. Most meat sample isolates also originated from broilers (n=161, 81% of all meat samples) and only a small amount was from pigs (14 isolates) and turkeys (24 isolates).

References in Supplementary Data

44. The European Commission. Commission implementing decision (EU) 2013/652 of 12 November 2013 on the monitoring and reporting of antimicrobial resistance in zoonotic and comensal bacteria (notified under document C (2013) 7145). In *Official Journal of the European Union*; The European Commission: Brussels, Belgium, 2013; Volume L 303, pp. 26–39
81. Prendergast, D.M.; O'Doherty, .; Burgess, C.M.; Howe, N.; McMahon, F.; Murphy, D.; Leonard, F.; Morris, D.; Harrington, C.; Carty, A.; et al. Critically important antimicrobial resistant Enterobacteriaceae in Irish farm effluent and their removal in integrated constructed wetlands. *Sci. Total. Environ.* **2021**, *806 Pt 3*, 151269.
82. Hooban, B.; Fitzhenry, K.; Cahill, N.; Joyce, A.; Connor, L.O.; Bray, J.E.; Brisse, S.; Passet, V.; Syed, R.A.; Cormican, M.; et al. A Point Prevalence Survey of Antibiotic Resistance in the Irish Environment, 2018–2019. *Environ. Int.* **2021**, *152*, 106466.
83. Ekhlas, D.; Sanjuán, J.M.O.; Manzanilla, E.G.; Leonard, F.C.; Argüello, H.; Burgess, C.M. Comparison of antimicrobial resistant Escherichia coli isolated from Irish commercial pig farms with and without zinc oxide and antimicrobial usage. *Gut Pathog.* **2023**, *15*, 8.
84. Wasyl, D.; Zając, M.; Lalak, A.; Skarżyńska, M.; Samcik, I.; Kwit, R.; Jabłoński, A.; Bocian, .; Woźniakowski, G.; Hoszowski, A.; et al. Antimicrobial Resistance in Escherichia coli Isolated from Wild Animals in Poland. *Microb. Drug Resist.* **2018**, *24*, 807–815.
85. Skarżyńska, M.; Zaja̧c, M.; Bomba, A.; Bocian, .; Kozdruń, W.; Polak, M.; Wia̧cek, J.; Wasyl, D. Antimicrobial Resistance Glides in the Sky—Free-Living Birds as a Reservoir of Resistant Escherichia coli With Zoonotic Potential. *Front. Microbiol.* **2021**, *12*, 656223.
